# Immunological reactions induced by bendamustine

**DOI:** 10.1186/2045-7022-4-S3-P56

**Published:** 2014-07-18

**Authors:** Maria-Jose Sanchez-Gonzalez, Jose Barbarroja-Escudero, Dario Antolin-Amerigo, Guadalupe Marco-Martin, Melchor Alvarez-De-Mon, Mercedes Rodriguez-Rodriguez

**Affiliations:** 1 Principe De Asturias University Hospital, Spain; 2 Principe De Asturias University Hospital, Allergy Department, Spain; 3 Principe De Asturias University Hospital, ESI Department, Spain

## Introduction

Bendamustine is being recently used as treatment of chronic lymphocytic leukemia (CLL) and B-cell non-Hodgkin lymphoma. There are just a few reported cases of adverse reactions to bendamustine so far, none of them with an allergological study.

## Method

### Patient 1

A 61-year old woman, diagnosed with B-cell CLL (B-CLL) received bendamustine, tolerating the first cycle. Three weeks later, 8 hours after the 1st dose of the 2nd cycle, she suffered throat discomfort, pruritus, hives, general erythema and facial swelling; which disappeared within 24 hours after treatment.

### Patient 2

A 61-year old man, diagnosed with B-CLL. Four years ago, he adequately tolerated bendamustine. One week after the first and well tolerated cycle, he received the second, and within 8-10 hours after the dose he developed a maculopapular exanthema, edema in arms and legs, along with desquamation of hands and feet, without residual lesions. Symptoms disappeared within the first two weeks.

### Patient 3

A 63-year old man, diagnosed with B-CLL. Three hours after the infusion of the first and second cycles of bendamustine, he had a generalized tremor and fever of 39℃. The symptoms ceased with acetaminophen 650mg. One month later, 3 hours after the 3rd cycle of bendamustine, he had generalized tremor and erythema, dizziness and body temperature of 39℃. Hypotension, paroxysmal atrial fibrillation and a mild renal failure were verified. The symptoms disappeared with acetaminophen 1gr and an adequate hydration.

## Results

We performed skin-prick-test (SPT) at 1 mg/ml and intradermal testing (IDT) at 0.001, 0.01, 0.1 and 1mg/ml, with immediate and delayed lectures (24h and 72h). As negative controls two B-CLL patients underwent SPT and IDT with negative results.

### Patient 1

SPT was negative. IDT at 0.1 and 1mg/ml were positive at 24h, being negative 72h later. The rest of the tests were negative.

### Patient 2

He had a negative SPT. The 24h lectures of the IDT were positive at 0.01, 0.1 and 1mg/ml, remaining positive 72h later. The rest of the tests were negative.

### Patient 3

The SPT and IDT, both immediate and delayed lectures, were negative.

## Conclusion

We report the first two cases of hypersensitivity to bendamustine with a positive result in the allergological study carried out, which demonstrated a delayed cutaneous hypersensitivity to bendamustine. We also report the first case of drug fever induced by bendamustine in clinical use, showing neither a type I nor a IV hypersensitivity mechanism to bendamustine.

